# Wearing the Beard

**Published:** 1881-12

**Authors:** 


### WEARING THE BEARD.

The fashion of the apostles, now almost universally restored among men, says the *Daily Advertiser*, was regarded with deep detestation by one of the merchant princes of Boston, whose name for three generations has been held in high honor. He had once made an appointment with a young artist, being himself confined to his house by infirmity of increasing years. When the artist appeared, his handsome face decorated then, as now, by a full beard, the gentleman gazed upon him with amazement for a moment, and then, forgetting his business and his infirmity, and with exceeding warmth of manner, ordered the young man out of his presence.

In 1850 a young man, who had contracted with a highly-respectable Pine street (New York) merchant for twelve months' service, was seized with a desire to let the hair grow on his upper lip. His employer treated it as a breach of contract, insisting that it would be a great damage to his trade for a clerk "to exhibit such a heathenish face."

This was the common feeling in banks, insurance companies, and like institutions. But it was especially fervent and intolerant in the church. One of the members of Rev. Dr. Bethune's church, in Brooklyn, having met with an accident which interrupted his usual habit of shaving for two or three weeks, found so little discomfort from the growth of that time that he decided to give it a further trial. When he appeared at church there was commotion among the good people, men and women. Several of them waited upon the doctor, after the service, to enlist him against this daring innovation. To their astonishment he had already gone over to the enemy, and quoted Scripture and the church fathers in support of the heresy. "But imagine," said one old lady, "a Chalmers or a Newton with such an unsightly growth!" The doctor gently answered, "When you come to example, my dear woman, imagine St. Paul or our Saviour without it, if you can!"

